# Influenza B Virus Receptor Specificity: Closing the Gap between Binding and Tropism

**DOI:** 10.3390/v16091356

**Published:** 2024-08-24

**Authors:** Caroline K. Page, Stephen Mark Tompkins

**Affiliations:** 1 Center for Vaccines and Immunology, University of Georgia, Athens, GA 30605, USA; cp29321@uga.edu; 2 Department of Infectious Diseases, University of Georgia, Athens, GA 30605, USA; 3 Center for Influenza Disease and Emergence Response (CIDER), University of Georgia, Athens, GA 30605, USA

**Keywords:** influenza B, FLUBV, receptor binding, glycosylation, sialic acids, virus–host interactions

## Abstract

Influenza A and influenza B viruses (FLUAV and FLUBV, respectively) cause significant respiratory disease, hospitalization, and mortality each year. Despite causing at least 25% of the annual disease burden, FLUBV is historically understudied. Unlike FLUAVs, which possess pandemic potential due to their many subtypes and broad host range, FLUBVs are thought to be restricted to only humans and are limited to two lineages. The hemagglutinins (HA) of both influenza types bind glycans terminating in α2,6- or α2,3-sialic acids. For FLUAV, the tropism of human- and avian-origin viruses is well-defined and determined by the terminal sialic acid configuration the HA can accommodate, with avian-origin viruses binding α2,3-linked sialic acids and human-origin viruses binding α2,6-linked sialic acids. In contrast, less is known about FLUBV receptor binding and its impact on host tropism. This review discusses the current literature on FLUBV receptor specificity, HA glycosylation, and their roles in virus tropism, evolution, and infection. While the focus is on findings in the past dozen years, it should be noted that the most current approaches for measuring virus–glycan interactions have not yet been applied to FLUBV and knowledge gaps remain.

## 1. Introduction

Influenza viruses belong to the family *Orthomyxoviridae* and are segmented negative-sense single-stranded RNA viruses containing eight gene segments: two encoding surface proteins and six encoding internal genes. The majority of human seasonal influenza virus burden and epidemics are attributed to influenza A and B viruses (FLUAV and FLUBV, respectively). While FLUAV is often regarded as the more severe influenza type, FLUBV contributes significantly to the overall disease burden and accounts for approximately a quarter of infections and hospitalizations annually [1,2]. Influenza B viruses have been circulating in humans for over 70 years. Ancestral strains of influenza B consisted of a single serotype which was first isolated in the 1940s [3]. In the 1970s, FLUBVs diverged into two antigenically distinct lineages known as Victoria and Yamagata [3,4,5,6]. Since the initial separation, these lineages have continued to diverge from each other as defined by the sequence homology of the hemagglutinin (HA) protein [6,7,8,9]. The HA protein along with the neuraminidase (NA) protein are the two major surface proteins expressed on influenza viruses and have historically been involved in reassortment events, which occur between the lineages. Although reassortment has significantly influenced the evolution of influenza B viruses, recent isolates since 2015 have followed independent evolutionary trajectories, with no reassortment between the lineages [10].

The two surface glycoproteins are the main targets of humoral immune responses and contribute to infection, pathogenicity, and receptor binding, albeit in different ways. The HA protein initiates infection by binding to cell surface receptors with glycans terminating in sialic acids, triggering endocytosis followed by pH-mediated endosomal fusion, leading to infection. The NA protein is a sialidase responsible for cleaving sialic acids on the surface of virions and infected cells, preventing virion aggregation during virus budding and release. While both surface glycoproteins play a critical role in the virus infection lifecycle, the HA protein has a dominant role in virus tissue tropism.

Glycans are molecules composed of carbohydrate units linked together through glycosidic bonds and are essential elements of influenza glycoproteins [11,12]. They play crucial roles in various biological functions including virus–host interactions, host immune responses, and protein folding. Influenza viruses frequently undergo genetic changes in their surface glycoproteins, particularly in the HA protein, which can alter glycosylation sites and thus affect the number of glycans expressed on the virus surface, impacting crucial biological functions. Both FLUBV lineages have evolved into subsets of antigenically distinct clades based on amino acid (AA) deletions and substitutions in the antigenic sites of the hemagglutinin protein [3]. For example, when Yamagata viruses first emerged, they contained a 2-AA deletion compared to Victoria viruses [6,13]. Later, these viruses reverted to a single AA deletion, and more recently, 3-AA variants have emerged, shifting the lineages from the now ancestral clade 2 to more contemporary clades 3 and 3A [6,10,13]. Similarly, Victoria viruses have undergone 2- and 3-AA deletions, resulting in a shift from clade 1B to 1A, with further diversification into subclades 1A.1 to 1A.4 [10,14]. These common AA deletions are located near the receptor-binding site, potentially impacting receptor-binding profiles as well as the antigenic character of the lineages.

Recently, increased divergence of evolutionary trajectories and unique seasonal infection dynamics have been observed with FLUBVs [10,15,16,17]. The evolutionary mechanisms of the Victoria lineage exhibit higher immune pressure on the HA protein, whereas the NA protein of Yamagata lineage viruses is evolving at a faster rate and driving its evolutionary dynamics [10]. Although the direct impact of this increased evolutionary pressure on virus–host interactions from contemporary lineage clades is not fully understood, the disappearance of the Yamagata lineage since the COVID-19 pandemic suggests there were significant changes in virus fitness [18,19]. This review will cover one aspect of that evolution, current knowledge of influenza B virus receptor specificity, and highlight the critical role of the viral glycoproteins, glycosylation, virus–host adaptations, and sialic acid distribution throughout the human body on virus tropism.

## 2. Influenza B Virus Receptor Specificity

**Figure 1:**
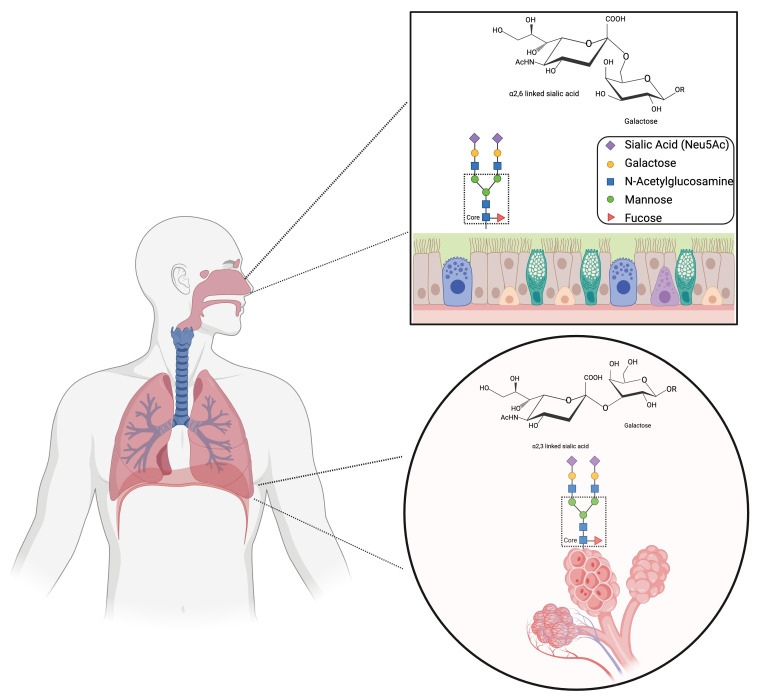
Distribution of influenza virus receptors in the human respiratory tract. Graphic in the top square depicts α2,6-sialic acid configuration glycans commonly found in the upper airways. Bottom circle depicts α2,3-linked sialic acid configuration glycans commonly found in the lower respiratory tract. Figure was created with BioRender.com.

The initial stage of viral infection begins with the binding of the influenza virus’ hemagglutinin protein to sialylated glycans present on the surface of host cells [20]. Sialic acids, a family of nine-carbon sugars, are commonly found at the terminal position of glycan chains on the surface of cells and soluble proteins. Among these, the prevalent sialylated glycans expressed on mammalian host cell surfaces are *N*-acetylneuraminic acid (Neu5Ac) and, notably absent in human hosts, *N*-glycolylneuraminic acid (Neu5Gc) [21]. The human respiratory tract, consisting primarily of epithelial cells, displays a wide spectrum of glycans predominantly terminating in α2,6-linked sialic acids (SAα2,6) in the upper respiratory tract and α2,3-linked sialic acids (SAα2,3) in the lower respiratory tract [22,23,24,25]. In the α2,6 configuration, a glycosidic bond is formed between the first carbon of the sialic acid and the sixth carbon of galactose, resulting in the structure of Neu5Acα2,6Gal. The same glycosidic bond is formed for the α2,3 configuration, except the first sialic acid carbon attaches to the oxygen on the third carbon of the sugar, resulting in Neu5Acα2,3Gal (Figure 1) [26]. For FLUAV, the preference of the HA for a specific terminal sialic acid linkage is a major determinant of species specificity, with avian-origin viruses binding SAα2,3 and mammalian-origin viruses binding SAα2,6 [27]. The spillover of avian influenza viruses into mammals does occur, however; in humans, this is believed to be from large exposure doses to the lower airways where SAα2,3 receptors are present [28,29]. An essential factor in the successful transmission of avian-origin influenza viruses to mammalian species is believed to be an expansion of sialic acid binding preference. Avian-origin viruses expand their binding from SAα2,3 to also include SAα2,6, enhancing their ability to infect epithelial cells in the upper-airway and facilitating more efficient transmission in humans and other mammals [24,29,30,31,32,33].

In contrast to the vast host range of FLUAVs, which includes birds, swine, equines, humans, and other mammals, FLUBVs exhibit a restricted host range, with humans being the only well-established natural reservoir [34]. There have been isolated FLUBV infections in seals, but these cases are infrequent and presumably due to spillover events from humans. Therefore, humans likely remain the main reservoir for FLUBVs and, consequently, are the drivers of virus evolution [35,36,37]. Due to the restricted host range, receptor specificity and virus–host interactions for influenza B viruses are distinct compared to FLUAV. While there is considerably less research defining receptor-binding profiles for FLUBVs compared to FLUAVs, it has been established that wild-type viruses from both lineages preferentially bind α2,6-linked sialic acids [30]. However, while Yamagata viruses appear to be restricted to α2,6-linked sialic acid binding, some Victoria lineage isolates exhibit broader binding specificity with the ability to bind both α2,3 and α2,6-linked terminal sialic acids [30].

The first studies assessing the receptor specificity of FLUBVs showed that the ancestral B/Lee/40 strain binds to Neu5Ac α2,6-sialic acids, as demonstrated by ganglioside thin-layer chromatography (TCL) assays [38]. Following this initial assessment of an ancestral FLUBV, Gambaryan et al. utilized solid-phase binding assays to define the receptor-binding profiles of egg-adapted and “authentic” (i.e., not egg-adapted) FLUAVs and FLUBVs. They observed the preferential binding of “authentic” human FLUAVs and FLUBVs to SAα2,6, while “authentic” avian FLUAVs and egg-adapted human FLUAVs and FLUBVs preferentially bound to SAα2,3 [39,40]. Continuing to explore the impact of egg adaptation, Lugovtsev and colleagues further defined the receptor-binding specificity of FLUBVs using glycan or carbohydrate microarrays. These high-throughput screening tools immobilize glycans in a spatially defined manner on slides to allow for the rapid analysis of receptor profiles [41]. Current arrays are expansive and can include complex *N*-glycans, *O*-glycans, symmetric and asymmetric monosaccharides, sialylated glycans, modified glycans, and more [42]. In studies utilizing glycan arrays to compare the receptor specificity of human and egg-adapted high-growth variants of B/Victoria/504/2000 (Yamagata lineage), the authors determined that FLUBVs could bind glycans terminally sialylated by Neu5Ac, but not Neu5Gc, and that longer oligosaccharide chains were preferred [43]. The parental human virus bound only SAα2,6, but the egg-adapted viruses gained SAα2,3 binding and then lost SAα2,6 binding during adaptation to high growth in eggs [43]. Moreover, the inner core, or central structural elements forming the backbone of the carbohydrate chain, were crucial determinants in the receptor–virus interaction [43]. The subsequent glycan array analysis of 53 FLUBVs isolated in Taiwan from 2001–2007 revealed that the FLUBV lineages have differential binding profiles, with Yamagata lineage viruses strictly binding to SAα2,6 while Victoria lineage viruses potentially bind to SAα2,3 in addition to SAα2,6 structures [30]. A small number of viruses bound sulfated glycans, although the importance of this was unclear. However, Bui and colleagues noted that following sialidase treatment to remove all SAα2,6 and SAα2,3 from red blood cells, the Victoria lineage virus B/Hong Kong/448799/2012 retained agglutination capabilities, further supporting the possibility that some FLUBVs may be able to utilize other receptors than SAα2,6 and SAα2,3 [44].

In 2013, Velkov summarized all influenza B virus receptor-binding data to date. He noted that HA may be given too much weight in defining FLUBV tropism, as data suggested that the receptor-binding pocket has the potential to bind both SAα2,6 and SAα2,3 [45]. However, Velkov also emphasized that additional studies are needed. To support this theory, a handful of studies building off that dataset have explored the determinants of receptor specificity [46,47,48]. These more recent publications have focused on molecular elements surrounding receptor-binding profiles, but studies exploring receptor-binding profiles from large datasets utilizing glycan arrays with new carbohydrate structures are lacking.

A persistent knowledge gap surrounding influenza B viruses is understanding why these viruses are restricted to human hosts. Specific characteristics of the human respiratory tract coupled with host adaptations can in part justify the restricted host range [49,50]. Influenza B virus’ requirements for proteolytic activation, pH stability, and temperature preference are optimally adapted to the conditions found in the human upper respiratory tract [50]. Receptor specificity in virus–host interactions has been extensively shown to play a role in host tropism for FLUAVs and can also partially explain host tropism for FLUBVs [24]. However, the lack of FLUBV receptor-binding studies encompassing a broad temporal and geographic selection of wild-type (i.e., not egg- or mouse-adapted) viruses, including contemporary viral isolates, renders it difficult to make broad conclusions regarding the determinants of FLUBV receptor binding and host tropism. Moreover, analysis using the most current tools and methods would support an improved understanding of the determinants of receptor specificity as well as the breadth of HA receptor ligands.

## 3. Contribution of Viral Glycoproteins to Receptor Binding

**Figure 2:**
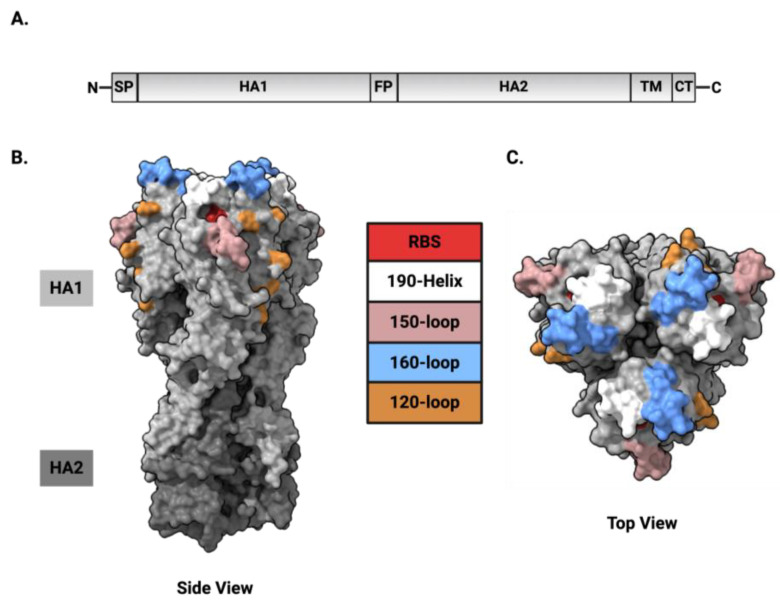
Influenza B virus hemagglutinin structure. (**A**) Schematic diagram of influenza B HA protein with the signal peptide (SP), HA1 subunit, fusion peptide (FP), HA2 subunit, transmembrane domain (TM), and cytoplasmic tail (CT), as depicted. (**B**) Side view (**C**) and top view of the HA protein with the four major antigenic sites and receptor-binding site (RBS) highlighted: RBS (red); 190-helix (white); 150-loop (pink); 160-loop (blue); and 120-loop (orange). Structures were modeled in ChimeraX. PDB 4FQM. Figure was created with BioRender.com.

Like the influenza A virus, the FLUBV HA protein is a trimer of identical subunits, with each subunit consisting of two polypeptide chains, HA1 and HA2, which form the globular head and stem region, respectively (Figure 2). The HA1 homotrimer constitutes the globular head and is primarily responsible for receptor binding. Within this subunit, there are four major antigenic regions: the 120-loop, 150-loop, 160-loop, and 190-helix. These regions are critical for the virus’s ability to infect cells and are major targets for host immune responses [31,47,51,52,53]. The more conserved HA2 subunit forms the stem region and contains a fusion peptide, which facilitates membrane fusion during viral entry [54]. The structural stability of the stem region, characterized by a trimeric coiled-coil structure and conserved residues, ensures the integrity of the trimeric HA complex.

The receptor-binding site (RBS) is a shallow depression at the top of the molecule and comprises parts of the 190-helix, 240-loop, and 140-loop of the HA1 monomer. Within the base of the RBS, there are four residues, Phe-95, Trp-158, His-191, and Try-202, which are conserved among all FLUBVs. These amino acids form the floor of the sialic acid binding pocket and account for many of the unique characteristics of the FLUBV RBS [31]. Specific amino acid residues within the hemagglutinin protein, especially within the RBS, play an essential role in receptor specificity and differ from structures defined with FLUAVs. For instance, FLUAVs possess Tyr-98, which stabilizes the base of the RBS via hydrogen bonds and enables high binding affinities to the sialic acid receptor [31]. In contrast, FLUBVs have Phe-95, which results in the loss of three hydrogen bonds crucial for stabilizing the RBS, resulting in markedly lower binding affinities to sialic acid for these viruses [47,55]. Studies mutating Phe-95 to Tyr in FLUBV demonstrated enhanced receptor-binding affinity between the virus and host [47]. Furthermore, mutations to residues Arg-162 and Asp-196 (R162M (Met) and D196Y (Tyr), respectively) expanded the range of glycans the parental virus could bind [43]. The crystal structure of B/Hong Kong/8/73 revealed position 196 to be within the RBS, unexpectedly implicating the residue in the receptor specificity of FLUBVs [31,46,52]. This will be discussed in depth later. Position 162, although not directly a part of the receptor-binding domain, is within close proximity of the binding site and showed similar effects on receptor binding as mutations at position 196 [43]. Comparing structures of the RBS for FLUBV to FLUAVs, Wang et al. noted that amino acid residue 237 for FLUBVs, corresponding to residue 222 for FLUAVs, also serves as a key determinant for HA binding to human sialic receptor analogs. Comparisons of influenza A and B HA crystal structures in complex with sialic acids showed that the hydrogen bonds interacting with galactose at this residue are responsible for the position in which the sugar fits into the binding pocket [31]. Notably, single amino acid changes near the RBS, occasionally associated with the loss of neutralizing antibody binding (i.e., antibody escape mutants), can also alter the structure and receptor-binding properties of influenza B viruses [46]. Together, these studies suggest that changes in the receptor-binding domain are influenced by immune pressure (i.e., antigenic drift) and can impact the receptor specificity of FLUBVs.

Once established that Victoria and Yamagata lineage viruses have differential binding preferences [30], a comparison of the crystal structures of the HA for the lineages contrasted their structural differences to determine how this may affect receptor binding [17]. Backbone variations in proximity to amino acid residues 165 and 180, particularly around the receptor-binding site, could influence FLUBV receptor specificity and partially explain some differences in the binding profiles between the lineages [17]. Analysis of specific antigenic residues within the HA indicated that from 1996 to 2007, Victoria viruses experienced stronger positive selection on the 190-helix compared to Yamagata viruses, which faced selective pressures on both the 120-loop and 190-helix from 1994 to 2005 [53,56]. After 2010, selective pressure for both lineages shifted primarily to the 120-loop [53]. These distinct selective pressures, especially around the 190-helix, which forms the receptor-binding site, potentially influenced the evolutionary development of differential binding profiles between the lineages. Further, AA deletions within the immunodominant HA1 residues 163–165, which are related to lineage differentiation, are located near the RBS and may also be essential in differences between receptor recognition between the lineages.

The neuraminidase protein of the influenza virus is a tetrameric glycoprotein consisting of a globular head domain containing the active site responsible for its enzymatic activity and a stalk region that forms the stem, anchoring the protein to the viral envelope [57]. The FLUBV NA protein is similar to the FLUAV NA protein and performs comparable functions for the virus’ replication cycle [57,58,59], including removing sialic acids from respiratory mucins and cilia to allow for efficient movement within the respiratory tract, catalyzing the cleavage of terminal SAs from virus receptors to facilitate viral fusion, and cleaving glycosidic linkages between terminal sialic resides from glycoproteins on infected host cells and newly formed viral particles, thereby promoting the infection process [12,58,60,61]. In addition to glycosylation sites, which will be detailed later, the NA protein also contains two calcium binding sites, one of which is located near the enzymatic site. These calcium-binding sites stabilize the protein and are essential in maintaining thermostability [58]. Similar to the HA protein, NA is under selective pressure (i.e., antigenic drift), and amino acid substitutions near the active site can alter the efficiency of influenza virus infection, resulting in the decreased binding of neuraminidase inhibitors such as oseltamivir and zanamivir [62]. Further, the desialidation of the HA protein by the NA enzymatic activity prior to translocation to the plasma membrane was essential for the hemadsorption properties of FLUBVs, exemplifying the importance of the NA–HA interactions in receptor binding [63]. FLUBV NA has yet to be shown to play a direct role in receptor binding as it has for some FLUAVs [64,65]. Nonetheless, there is a critical dynamic balance between HA:NA that must be maintained for receptor binding to occur [66,67,68].

## 4. Influenza B Glycosylation

**Figure 3:**
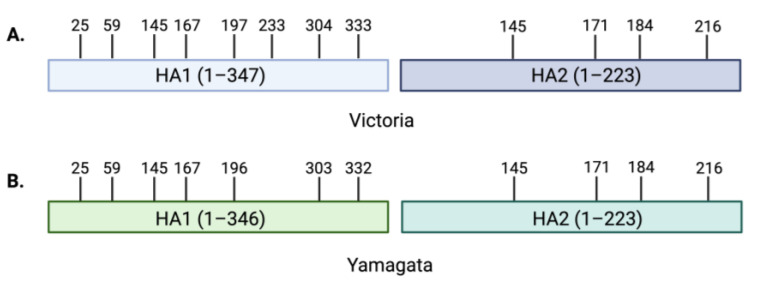
Potential N-glycosylation sites on the HA1 and HA2 of recent Victoria and Yamagata lineage viruses. (**A**) Victoria lineage HA glycosylation sites on the HA1 subunit (light blue) and HA2 subunits (dark blue). (**B**) Yamagata glycosylation sites on HA1 (light green) and HA2 (dark green). Figure was created with BioRender.com.

Glycosylation is a common post-translational modification in which sugar molecules, known as glycans, are covalently attached to specific amino acids such as Asn for N-linked glycans and Ser or Thr for O-linked glycans [12]. The glycans are transferred onto proteins by a group of enzymes called glycosyltransferases while the proteins move through the ER and Golgi [29,69]. The globular head and stem region of the FLUBV HA proteins carry N-linked oligosaccharides with 10–12 glycosylation sites on both the B/Victoria and B/Yamagata lineage viruses (Figure 3) [12]. In the stem region of the HA, conserved glycosylation sites are essential in stabilizing and enhancing the trimerization of the protein [12,70,71]. On the globular head, glycosylation sites can vary between each lineage and have been shown to play a direct role in the receptor-binding profiles for FLUBVs [39,40,72]. Proper glycosylation of the HA is essential to protein function and can significantly impact host–pathogen interactions by altering receptor binding, pathogenicity, and antigenicity [68,73,74].

The significance of HA-head glycosylation patterns on receptor binding is exemplified by the common loss of a glycosylation site at amino acid residue 196 following the propagation of FLUBVs in embryonated chicken eggs, which affects the receptor-binding properties [39,72,73,74,75]. The glycosylation motif Asn-X-Thr/Ser enables asparagine(N)-linked glycosylation. Studies as early as 1985 demonstrated that the serial passage of influenza B viruses in MDCK cells maintained the naturally occurring N-linked glycosylation motif at amino acid 196. However, the serial passage of FLUBV in embryonating chicken eggs resulted in mutations at Asn-196, abolishing the glycosylation site [72,73,74]. Viruses retaining the glycosylation motif at position 196 replicate well in Madin-Darby Canine Kidney (MDCK) cells, which express both SAα2,6 and SAα2,3 receptors, but replicate poorly in eggs, which express mostly SAα2,3 [15,73]. In 1997 Gambaryan et al. demonstrated for the first time, using a competitive solid phase assay bearing 3′- and 6′-sialyllactose moieties (3-SL and 6-SL, respectively), that the loss of the Asn-196 glycosylation site in egg-grown viruses affects the receptor-binding phenotype. Specifically, egg-grown viruses exhibited expanded binding abilities to both 6-SL and 3-SL, whereas cell-grown viruses bound only to 6-SL [40]. Complexed structures of influenza B HA from B/Hong Kong/8/1973 (B/HK) with sialic acid moieties suggest that the glycan attached to Asn-196 interferes structurally with the binding of SAα2,3, which is abundant in chicken eggs [31]. The restricted binding profile was shown to be detrimental to the growth of the viruses maintaining glycosylation at Asn-196 in eggs, demonstrating that egg adaptation allows for strong selective pressure on this glycosylation site to allow for a broader binding to avian SAα2,3 receptors and enhanced viral replication [73,74]. Furthermore, using FLUBV-immune ferret anti-sera, Chen et al. demonstrated that changes to the glycosylation at amino acid residue 196 altered virus antigenicity. They found that an Arg-141 was essential for stabilizing the glycosylation site following replication in eggs, which has implications for establishing egg-adapted vaccine viruses [73].

While the effects of egg adaption on the glycosylation site at amino acid 196 have demonstrated the significance of this glycan on FLUBV receptor-binding profiles, understanding evolutionary pressures at this residue in naturally occurring (non-egg adapted) viruses is essential, as well. There are differential selective pressures on the FLUBV lineage viruses, and recently the evolutionary trajectories of the Victoria and Yamagata lineages were shown to be diverging. Considering the direct role that HA plays in receptor binding, differences in the *N*-glycosylation patterns on the Victoria and Yamagata HA surface could, in part, explain the differences in binding profiles between the lineages [30]. Models of the distinct lineage mutations within the HA protein suggest that the differential selective pressures of Victoria compared to Yamagata viruses on the 194–196 glycosylation site in authentic viruses may affect the differences in receptor-binding profiles [17]. The Asn-196 *N*-glycosylation site is highly conserved among Yamagata viruses and, in contrast, Asn-196 occurs at a lower frequency for Victoria viruses [17]. Considering this glycosylation site is proximal to the RBS, a functional consequence of losing the glycosylation would be opening the RBS to allow for the accommodation of not only SAα2,6 but also SAα2,3 receptors [17]. In turn, the distinct selective pressures on the wild-type Victoria and Yamagata viruses could explain the differences in receptor-binding profiles that have been observed between the lineages. In addition to the *N*-glycosylation site at position 196, both lineages share a potential glycosylation site at amino acid 145 and Victoria lineage viruses also have a glycosylation site at position 230 (Figure 3) [76]. However, the distance of amino acids 145 and 230 from the receptor-binding domain likely limits the influence of glycosylation at these sites on receptor-binding differences [17].

In contrast to the eight N-glycosylation sites for the FLUAV NA tetramer, there are 4–5 potential glycosylation sites on influenza B neuraminidase [12,68]. The *N*-glycan at Asn-146 is conserved across all influenza viruses and has been shown to have effects on neurovirulence and NA enzymatic activity for FLUAVs [58,77]. The role of glycosylation of NA is less understood than that of HA; however, it is important in maintaining the function and sialidase enzymatic activity of the protein [12]. Additionally, glycosylation of the NA protein stabilizes the structure and reduces its conformational flexibility, which affects receptor binding by impacting the critical NA:HA balance [78]. Traditionally, reassortment between the lineages HA and NA proteins may have rendered this balance equivalent. However, with the lack of reassortment and increased evolutionary divergence of contemporary FLUBVs, the role of this HA:NA balance should be further explored. Additional studies are also needed to fully characterize the effects of glycosylation patterns on the FLUBV NA protein and receptor-binding properties of the viruses.

## 5. Host Cell Glycans and FLUBV Clinical Manifestations

The distribution of sialic acids on host cell surfaces plays a crucial role in determining the host tropism and clinical manifestations of influenza infection. The types and distribution of sialic acids vary among different tissues and species, influencing which cells and tissues the virus can potentially infect, thereby affecting the severity and symptoms of infection [30]. Avian influenza viruses, for example, typically bind to sialic acids with α2,3-linkages, predominantly found in the lower respiratory tract of humans, potentially leading to more severe respiratory illnesses such as in the case of human H5 infections [79]. In contrast, human viruses prefer α2,6-linked sialic acids, common in the upper respiratory tract, enabling respiratory transmission and potentially resulting in milder clinical symptoms [32,33,80,81]. While less is known about how the distribution of sialic acids in the respiratory tract fully affects infection and disease severity for influenza B viruses, there is evidence suggesting that FLUBV receptor specificity may influence host tropism, age distribution of infection, and disease pathogenesis.

### 5.1. Children vs. Adults

The frequency of infection between Victoria and Yamagata lineage viruses varies with age, with Victoria lineage viruses predominantly infecting younger populations and Yamagata lineage viruses more commonly infecting older populations [1,17,82,83,84]. One hypothesis for the age distribution disparities between FLUBV lineage infections focuses on differences in receptor-binding profiles between the lineages and the distribution of host cell glycans in human airways [1,17]. There are subtle variations in the expression and prevalence of SAα2,6 and SAα2,3 in the epithelial cells lining the respiratory tract of children compared to adults, with SAα2,3 receptors being more prominent in children [17,22,24]. The SAα2,3 expression in children, combined with Victoria lineage viruses often possessing expanded receptor-binding capabilities to include SAα2,3 in addition to SAα2,6, compared to Yamagata viruses (binding only SAα2,6), could account for the increased frequency of Victoria lineage infection in children. Interestingly, influenza B infections with viruses that lose the *N*-glycosylation site at 196 on the HA are more common in younger children [17]. Therefore, changes in HA glycosylation that expand FLUBV binding to both SAα2,3 and SAα2,6 receptors, coupled with differences in sialic acid expression between children and adults, may help explain the differential age distribution of the Victoria and Yamagata lineage FLUBV infections. However, data on sialic acid expression in tissues from children is limited and requires additional analysis using state-of-the-art approaches to define the tissue glycome. Therefore, studies confirming this hypothesis are limited and need to be further explored.

### 5.2. Upper and Lower Respiratory Tract Infections

In addition to differences in the age distribution of FLUBV lineage infections, there are also links between the receptor-binding profiles in FLUBVs and the clinical presentations in patients. Specifically, the SAα2,3 binding of some Victoria lineage viruses may enable the infection of different tissues based upon sialic acid expression on tissues throughout the body [30]. Studies with tissue explant cultures of human bronchus and lungs showed that FLUBVs spanning multiple evolutionary clades are capable of infecting ciliated, club, goblet, and basal cells, like human FLUAVs, demonstrating similar tissue tropism [44]. In adult humans, the upper respiratory tract expresses higher levels of SAα2,6 compared to the lower respiratory tract, which contains more SAα2,3 [22,24,25]. In the upper respiratory tract, SAα2,6 is expressed on both ciliated and non-ciliated cells but not on alveolar cells found in the lower respiratory tract [85]. In contrast, SAα2,3 is primarily found on alveolar cells in addition to low levels on ciliated epithelial cells in the upper respiratory tract [85]. Influenza B viruses exhibit higher replication in human bronchial explants compared to lung explants, which is consistent with HA binding patterns and sialic acid distribution between these organs [44]. Interestingly, FLUBVs with dual SAα2,6 and SAα2,3 binding specificities have been linked to greater frequencies of lower respiratory tract symptoms, while the SAα2,6-only binding viruses are generally restricted to upper respiratory tract infections [30].

### 5.3. Gastrointestinal Symptoms

Both α2,6 and α2,3-linked sialic acids are present on cells lining the gastrointestinal (GI) tract, but their distribution can vary depending on the specific region—whether it is the stomach, small intestine, large intestine, or colon—as well as the age, diet, and health of the individual [86]. Typically, there is a higher expression of SAα2,3; however, limited studies fully detail the distribution of sialic acids in the GI tract of humans and their connection to influenza virus infection mainly because the virus is transmitted through a respiratory route [87]. Compared to respiratory symptoms, vomiting and diarrhea are less common clinical signs associated with influenza virus infections and are more frequently associated with influenza B infections than with influenza A [88,89]. Furthermore, Victoria viruses have been notably linked to gastrointestinal symptoms when compared to Yamagata viruses [90]. In addition to increased symptoms in the lower respiratory tract of patients infected with viruses that bind to both SAα2,3 and SAα2,6 glycans, gastrointestinal symptoms were also associated with the dual binding. Overall, severe illness is increased in dual-binding viruses compared to viruses that bound SAα2,6 only [30].

### 5.4. Conjunctivitis

The occurrence of conjunctivitis following influenza B virus infection, though rare, underscores the importance of understanding the distribution of host cell sialic acids and how receptor-binding preferences may influence these infections [88,91,92,93]. While relatively little is known about the distribution of sialic acids in the human eye, both SAα2,3 and SAα2,6 have been identified in conjunctival epithelial cells [94,95,96]. Furthermore, there is a predominance of SAα2,3 on the ocular surface, which may explain the limited susceptibility of human influenza A viruses that preferentially bind SAα2,6. However, avian influenza viruses, which preferentially bind SAα2,3, have been shown to cause clinical signs such as conjunctivitis [97,98,99,100,101,102,103,104]. Notably, in 2001, there was a documented case of conjunctivitis following an accidental exposure to B/Shangdong/07/97 [91]. It is important to recognize the potential for FLUBV infection and replication in the eye and to better understand the role of sialic acid distribution in tissue tropism [85]

### 5.5. Neurotropism

Neurological symptoms during influenza infection are considered rare but have been reported in FLUBV infections [105,106,107,108]. Neurological symptoms are most commonly observed in young children, elderly, or immunocompromised individuals. These symptoms often result from severe systemic illness, secondary complications, or inflammatory responses. Although the neurotropism of FLUBV has not been directly linked to receptor specificity, it may influence the distribution of the virus throughout the body and contribute to the overall severity and complications of the disease. While little is known regarding the specific factors that lead to neurological disease, it underscores the importance of further investigating the connection between receptor specificity and disease severity.

## 6. Conclusions and Future Perspectives

In contrast to the extensive literature exploring receptor specificity of various subtypes and evolutionary clades of FLUAV using modern and advanced techniques, there is relatively limited information on the receptor specificity of influenza B viruses [30,38,39,40,43,74,109,110]. Moreover, much of the data for FLUBV has relied on less refined methods such as solid-phase ELISA and ganglioside TLC assays, while more sophisticated and biologically relevant techniques like glycan arrays, biolayer interferometry, erythrocyte remodeling, and surface plasmon resonance are now available [111,112,113]. Glycobiology has seen significant advancements in microarray technology, allowing for arrays that better represent the human cell surface for multiple tissues and provide more detailed information about receptor binding and host tropism beyond SAα2,3 and SAα2,6 binding profiles [111]. Although a handful of studies have assessed FLUBV receptor specificity using early glycan arrays [30], there have been no comprehensive assessments of influenza B HA receptor specificity using modern tools. Furthermore, the most robust study to date exploring the receptor specificity of FLUBVs via glycan array was limited by the availability of complex branched biologically relevant structures at the time and focused only on a set of clinical isolates from Taiwan between 2001–2007 [30]. A comprehensive analysis of ancestral, Victoria, and Yamagata lineage virus receptor specificity evolution is needed, such that virus tropism can be integrated into our understanding of FLUBV evolution. The recently described increased rates of evolution and endemic activity seen with contemporary influenza B isolates could be mediated, in part, by changes in tropism for the different lineages [10,17].

Although there is a growing interest in understanding the determinants of host tropism for FLUBVs [49], questions remain regarding their restricted host range. One potential hypothesis explaining the limited host range revolves around the weak receptor-binding capacity of FLUBV HA proteins compared to FLUAV. This suggests that an optimal density of cell-surface sialic acid receptors with the proper structure and configuration may be required for efficient and successful influenza infection [47,114]. If this optimal distribution is only achieved in the human respiratory tract, it could, in part, explain the host restriction of FLUBVs to humans. Interestingly, a study using a chimeric live-attenuated influenza B virus expressing an avian H5 hemagglutinin protein was unable to infect chickens, demonstrating the requirement of multiple species-specific adaptations beyond receptor specificity required for the successful transmission of influenza B viruses from humans to avian hosts [115].

Here, we summarized current knowledge about the structural characteristics of FLUBV HA proteins and how they interact with the neuraminidase to determine receptor specificity and the strength of binding. Recent studies have defined the effects of glycosylation patterns on receptor binding for egg-adapted and wild-type FLUBVs, suggesting that differing evolutionary pressures on key antigenic sites of the hemagglutinin proteins of Victoria and Yamagata lineages contribute to their differential receptor-binding profiles. A comparison of clinical disease manifestations and the receptor-binding profiles of FLUBVs has provided insight into disease pathogenesis based on tissue tropism influenced by HA receptor specificity and sialic acid distribution on host cells. These studies also suggest that the expanded sialic acid binding profile of Victoria lineage viruses is associated with lower respiratory and gastrointestinal tract infections, a younger host-age range, and overall, greater clinical disease severity compared to Yamagata lineage viruses. While notable strides have been made in our understanding of virus–host interactions for FLUBVs, significant knowledge gaps remain. Further studies are needed to define the mechanisms of the heightened endemic activity and continued divergence of FLUBVs over the past decade and the potential displacement of Yamagata lineage viruses from human circulation following the COVID-19 pandemic.

## Data Availability

No new data were created or analyzed in this study. Data sharing is not applicable to this article.
